# No Difference in the Phenotypic Expression of Frailty among Elderly Patients Recently Diagnosed with Cancer vs Cancer Free Patients

**DOI:** 10.1007/s12603-019-1293-8

**Published:** 2019-11-08

**Authors:** K. El Haddad, Y. Rolland, S. Gérard, L. Mourey, S. Sourdet, B. Vellas, E. Stephan, G. Abellan Van Kan, P. De Souto Barreto, L. Balardy

**Affiliations:** 1 Gérontopôle, Department of Internal Medicine and Geriatrics, Toulouse University Hospital, Toulouse, France; 2 INSERM U1027, University of Toulouse III, Toulouse, France; 3 Medical oncology department, Claudius Régaud Institute — Oncopôle, Toulouse Cancer University Institute (IUCT-O), Toulouse, France; 4 Department of Geriatric Medicine, Saint Georges Hospital University Medical Center, Beyrouth, Lebanon; 5 Institut du Vieillissement, Gérontopôle, Université Toulouse III Paul Sabatier, 37 Allées Jules Guesde, 31000 Toulouse, France

**Keywords:** Geriatric oncology, elderly, frailty phenotype, frailty index

## Abstract

**Objectives:**

To examine frailty determinants differences in patients with a recent diagnosis of cancer compared to non-cancer patients among older adult. Revealing those differences will allow us to individualize the exact frailty management in those patients diagnosed with cancer.

**Design:**

This is an observational cross-sectional, monocentric study.

**Setting:**

Patients were evaluated at the Geriatric Frailty Clinic (GFC), in the Toulouse University Hospital, France, between October 2011 and February 2016.

**Participants:**

1996 patients aged 65 and older were included (1578 patients without cancer and 418 patients with solid and hematological cancer recently diagnosed).

**Measurements:**

Frailty was established according to the frailty phenotype. The frailty phenotype measures five components of frailty: weight loss, exhaustion, low physical activity, weakness and slow gait. Frailty phenotype was categorized as robust, pre-frail and frail.

**Results:**

In a multinomial logistic regression, cancer, compared to the non-cancer group, is not associated with an increased likelihood of being classified as pre frail (RRR 0.9, 95% CI [0.5; 1.6], p 0.9) or frail (RRR 1.2, 95% CI [0.7; 2.0], p 0.4) rather than robust. When considering each Fried criterion, a significant higher odd of weight loss was observed in older patients with cancer compared to the non-cancer patients (OR 2.3, 95% CI [1.8; 3.0], p <0.001) but no statistically significant differences was found among the four other Fried criteria. Sensitivity analysis on the frailty index showed that cancer was not associated with a higher FI score compared to non-cancer (β 0.002, 95%CI [-0.009; 0.01], p 0.6).

**Conclusion:**

In this real-life study evaluating elderly patients with and without cancer, we didn’t confirm our hypothesis, in fact we found that cancer was not associated with frailty severity using both a phenotypic model and a deficit accumulation approach. Cancer may contribute, at least additively, to the development of frailty, like any other comorbidity, rather than a global underlying condition of vulnerability.

## Introduction

Frailty has been incorporated in geriatric oncology to tailor the medical management accordingly (1). It reflects the loss of biological reserves, arising from cumulative deficits in several physiological systems and resulting in a diminished resistance to stressors (2, 3). Since cancer per se and the corresponding treatment are stressors that may aggravate those biological reserves, their assessment of frailty among cancer patients is primordial.

Many articles attempted to study different frailty screening or evaluation methods and their clinical relevance in the assessment of older patients with cancer (4, 5). Furthermore, studies showed that frailty emerges in geriatric oncology as a predictor for an increased risk of chemotherapy intolerance, postoperative complications and mortality6 and was associated with negative outcomes such as an increase physical decline and poor functioning (5).

However, there is no clear identification of the exact frailty phenotype of older cancer patients and how they differ between frail elder patients without cancer. Although, from a clinical viewpoint, it is expected that older cancer patients would have weight loss (7), one of the components of the frailty phenotype (8), more often than non-cancer patients, this has never been investigated in a sample of patients looking for the same frailty care service. This is also true for the other components of the frailty phenotype. Since cancer treatment may constitute an aggressive stressor to the frail elderly (2), it is important to identify if a condition of increased frailty severity that can be found after cancer treatment is due to the treatment itself or due to an increased frailty level before treatment starts. To the best of our knowledge, there is few if any article that tackle the association of cancer on the loss of these physiological reserves, in other words the difference of frailty phenotype between cancer patients and cancer-free patients.

Our hypothesis is that frailty determinants differs between cancer patients and cancer free patients. Revealing those differences will allow us to individualize the exact frailty management in those patients diagnosed with cancer. In the present work, we aim at providing initial data on the differences of frailty determinants among recently diagnosed cancer patients not receiving yet the treatment compared to non-cancer patients in a large sample of older adults looking for a frailty ambulatory healthcare service.

## Methods

### Sample population

In this observational cross-sectional, monocentric study, we reviewed the electronic medical records of all patients who underwent medical evaluation at the Geriatric Frailty Clinic (GFC), in the Toulouse University Hospital, France, between October 2011 and February 2016. Patients were referred by their primary care physician for frailty assessment or by their oncologist for care plan management including pre-cancer treatment. The methods of this study have been previously described in detail by Tavassoli et al. (9).

### Data collection

A total of 1996 patients aged 65 and older were included in this study (1578 patients without cancer and 418 patients with cancer). Data extracted from patient’s medical information included sociodemographic (living environment, marital status, educational level), anthropometric and clinical data (medical and surgical history, and current treatment). Charlson Comorbidity Index (10) (CCI) was also assessed but modified by omitting the following comorbidities in our analysis: tumor, metastasis, leukemia and lymphoma in order to limit confounding effect. Patient’s degree of disability using the basic Activities of Daily Living scale (ADL) (11) and Body Mass Index (BMI) (12) were also assessed. Furthermore, evaluation of four geriatric domains were also extracted from the patients’ medical information which include; the Mini-Mental State Examination (MMSE) for cognition testing (13), the Short Physical Performance Battery (SPPB) for physical function (14), the Mini Nutritional Assessment (MNA) (15) as nutritional evaluation and the visual and auditive acuity for evaluating sensory capacity. Sensory evaluation was assessed using visual and hearing capacities. Visual capacity was evaluated using the Snellen decimal chart for distant vision, the Parinaud chart for near vision and the Amsler grid for macular degeneration assessment. We considered normal vision, patients who have normal distant vision (distant visual acuity ≥20/40), normal near vision (Parinaud 2) and negative Amsler grid testing (no scotoma and/or metamorphopsia) in both eyes. Hearing capacity was assessed by the Hearing Handicap Inventory for the Elderly Screening tool (HHIE-S) (16).

### Outcome measure

Frailty was established according to the frailty phenotype (8). The frailty phenotype consists of five components: weight loss, exhaustion, low physical activity, weakness and slow gait. Score ranges from 0 to 5. Subjects were categorized as: a score of 0 for robust people, a score of 1–2 for pre-frail, and a score of 3 or more for frail.

### Statistical analysis

Descriptive statistics were performed to present tumor characteristics among cancer patients as well as characteristics between patients with and without cancer. Results were obtained using means (mean ± SD) or absolute numbers (%) as appropriate. Bivariate analysis was undertaken using the Chi-2 test or the Fisher’s exact test for categorical variables and student t-test or Wilcoxon rank sum test for continuous variables, as appropriate. In addition to age and sex, the variables living place, education and Charlson Comorbidity Index (CCI) were included into a multivariate analysis as confounders. A multinomial logistic regression analyzed the association of cancer/non-cancer with the frailty phenotype (ie, robust, pre-frail, and frail). Binary logistic regressions were performed separately for each of the five frailty criteria (weight loss, exhaustion, low physical activity, weakness and slow gait) composing the frailty phenotype. Statistical significance was set at p <0.05. Multiplicity in the separate analyses per frailty component (five different models) was taken into account by using Bonferroni adjustment, with p-value set at p <0.01. All analysis was performed with STATA version 14.2 (College Station, TX: StataCorp LP).

#### Sensitivity analysis

Sensitivity analysis was done for controlling for the possibility that non-cancer patients may have a worse health status than cancer patients and that may not be captured by a phenotypic model, which is restrict to 5 items, by homogenizing the 2 groups. A multiple linear regression examined the association of cancer/non-cancer with the continuous frailty index (FI) (17). The latter was constructed with 34 variables including comorbidities, degree of disability and nutritional assessment. Frailty index (FI) was the total deficits as a proportion of those counted for each patient.

## Results

Among those 1996 patients (median age 82.9 [78.7; 86.9]), 418 were diagnosed with cancer. Mostly diagnosed cancers were digestive, hematologic and urologic cancer (29.6%, 18.9% and 18.4%, respectively). Cancer characteristics among those 418 patients are described in table 1. Cancer and non-cancer patients’ characteristics are shown in table 2. Weight loss was the only frailty criterion difference between the two groups with more patients having weight loss in the cancer groups compared to that of the cancer-free group (42.8% vs 23.1%, respectively). Results from the multivariate analysis are reported in table 3. In summary, we found that cancer, compared to non-cancer, is not associated with an increased likelihood of being classified as pre-frail or frail rather than robust (Relative Risk Ratio (RRR) 0.9, 95% CI [0.5; 1.6] p 0.9 for the pre-frail group and RRR 1.2, 95% CI [0.7; 2.0], p 0.4 for the frail group). Sensitivity analysis on the frailty index showed that cancer was not associated with a higher FI score compared to non-cancer (β 0.002, 95%CI [-0.009; 0.01], p 0.6).

**Table 1:** Cancer characteristics among the 418 cancer patients

**Measures**	**Mean ± SD, median [p25 p75], N (%)**
Age (in years)	82.7 ± 5.5
Female	201 (48.1)
Nb of pts benefiting from social support (n =416)	244 (58.6)
Solid Cancer	
- Dermatological*	339 (81.1)
- Digestive	10 (2.4)
- Gynecological¥	124(29.7)
- HEENT	70 (16.8)
- Pulmonary	24 (5.7)
- Urologic	18 (4.3)
Hematological Cancer	
- AML	79 (18.9)
- CLL	1 (0.2)
- Lymphoma	34 (7.9)
- Myelodysplasia	23 (5.5)
- Myeloma	8 (1.9)
Metastatic solid tumor (n = 179)	
- Present	73 (38.8)
- Absent	106 (60.1)
Cancer stage	
- Stage I	155 (37.0)
- Stage II	75 (17.9)
- Stage III	109 (26.0)
- Stage IV	79 (18.9)
G8 score (n=397)	12 [9.5; 13.5]
Charlson Comorbidity Index**	4 [4; 5]

*Patients with dermatological cancer include SSC (1.2%), BCC (0.24%), Melanoma (0.7%) and spinocellular (0.24%), ¥ 51 patients have breast cancer in the gynecological group, ** Charlson Comorbidity Index11 (CCI) was modified by omitting the following comorbidities in our analysis: tumor, metastasis, leukemia and lymphoma in order to limit confounding effect.

**Table 2:** Bivariate analysis of patients’ characteristics between cancer-free and cancer groups

	**Patients without Cancer, n = 1578**	**Patients with Cancer, n = 418**	**Total number of patients, n = 1996**	**p**
Age (years)	82.9 [78.5; 87.0]	82.8 [79.3; 86.4]	82.9 [78.7; 86.9]	0.9
Female (N, %)	1080 (68.4)	201 (48.0)	1281 (64.1)	<0.001
Marital status (N, %)				0.01
Widowed	713 (45.6)	161 (38.5)	874 (44.1)	
Married	612 (39.1)	203 (48.5)	815 (41.1)	
Single	95 (6.0)	21 (5.0)	116 (5.8)	
Divorced	139 (8.9)	32 (7.6)	171 (8.6)	
Living place (N, %)				0.001
Home — alone	912 (58.5)	284 (68.2)	1196 (60.6)	
Home with partner	552 (35.4)	110 (26.4)	662 (33.5)	
Assisted living	46 (2.9)	6 (1.4)	52 (2.6)	
Nursing home	47 (3.0)	16 (3.8)	63 (3.2)	
Education (N, %)				<0.001
No education	69 (4.6)	30 (7.2)	99 (5.1)	
Primary school	537 (35.7)	202 (48.6)	739 (38.5)	
Middle school	366 (24.3)	69 (16.6)	435 (22.6)	
High school	232 (15.4)	45 (10.8)	277 (14.4)	
University	300 (20.0)	69 (16.6)	369 (19.2)	
Activity of Daily Living (ADL)	6 [5; 6]	6 [5; 6]	6 [5; 6]	0.7
CCI*	4 [4; 5]	4 [4; 5]	4 [4; 5]	<0.001
MMSE	26 [21; 28]	26 [23; 29]	26 [22; 29]	0.1
SPPB	8 [5; 10]	8 [5; 10]	8 [5; 10]	0.4
MNA	25 [22; 27]	23 [20; 25]	24.5 [21.5; 26.5]	<0.001
Audition HHIE-S** (N, %)				0.8
Normal	835 (52.9)	224 (53.6)	1059 (53.0)	
Mild to moderate hearing loss	531 (33.6)	143 (34.2)	674	(33.7)
Severe hearing loss	212 (13.4)	51 (12.2)	263 (13.1)	
Vision ¥ (N, %)	0 (0)	147 (35.1)	147 (7.3)	<0.001
Frailty Index	0.17 [0.11; 0.26]	0.19 [0.11; 0.29]	0.17 [0.11; 0.27]	0.057
Fried phenotype (N, %)				0.1
Robust	149 (9.5)	33 (7.9)	181 (9.2)	
Pre-frail	632 (40.8)	155 (37.1)	787 (40.0)	
Frail	766 (49.5)	229 (54.9)	995 (50.6)	
Fried criteria				
Weight loss	363 (23.1)	179 (42.8)	542 (27.3)	<0.001
Exhaustion	759 (49.1)	214 (51.5)	973 (49.6)	0.3
Low physical activity	834 (53.1)	200 (47.8)	1034 (52.0)	0.1
Weakness	741 (52.4)	180 (53.2)	921 (52.6)	0.9
Slow gait	360 (40.2)	69 (33.9)	429 (39.0)	0.1

*CCI; Charlson Comobidity Index was calculated by removing the following comorbidities: cancer, metastasis, leukemia and lymphoma. **HHIE-S; Hearing Handicap Inventory for Elderly-Screening: score defined as: 0–8 normal, 10–24 mild to moderate hearing impairment, 26–40 severe hearing loss. ¥ Vision Visual capacity was evaluated using the Snellen decimal chart for distant vision, the Parinaud chart for near vision and the Amsler grid for macular degeneration assessment. We considered normal vision, patients who have normal distant vision (distant visual acuity ≥20/40), normal near vision (Parinaud 2) and negative Amsler grid testing (no scotoma and/or metamorphopsia) in both eyes.

**Table 3:** Multivariate analysis of Frailty phenotype and frailty criteria in cancer vs cancer-free groups

	**RRR**	**95% CI**	**p**
Outcome			
Frailty Phenotype **			
Pre-Frail*			
Cancer	0.9	[0.5; 1.6]	0.9
Frail*			
Cancer	1.2	[0.7; 2.0]	0.4
	**OR**	**95% CI**	**p**
Outcome			
Fried criteria			
Weight Loss¥			
Cancer	2.3	[1.7; 3.1]	<0.001
Exhaustion¥			
Cancer	1.06	[0.8; 1.4]	0.6
Low physical activity¥			
Cancer	0.8	[0.6; 1.0]	0.1
Weakness¥			
Cancer	1.07	[0.8; 1.4]	0.6
Slow gait¥			
Cancer	0.8	[0.5; 1.2]	0.2

Note: RRR; Relative Risk Ratio, OR; Odd ratio, IC95%; 95% confidence interval.

** Outcomes pre-frail and frail phenotype are compared to the robust phenotype. * Variable associated with a p value of < 0.05 was considered significative. ¥ Variables associated with a p value of < 0.01 was considered significative after using Bonferroni adjustment.

## Discussion and conclusion

To the best of our knowledge, there is no clear identification of the exact frailty phenotype of older cancer patients and how they differ between frail elder patients without cancer. Our present article is one of the first to examine if recently diagnosed cancer is associated with patient’s frailty status beyond age and sex using the phenotype model. In fact, this model is one of the most evidence-based approaches to the identification of frailty (2) but was operationalized by Fried et al. using data from the Cardiovascular Health Study with no cancer patient at inclusion (8). In this real-life study evaluating elderly patients with and without cancer, we didn’t confirm our hypothesis, in fact we found that cancer was not associated with frailty severity using both a phenotypic model and a deficit accumulation approach. As expected, cancer was associated with a 2.3-fold increase in the probability to have weight loss. No other component of the frailty phenotype differed between patients with cancer and those without.

Our findings showed that frailty is distinct from, but overlapping with the cancer. This latter may contribute, at least additively, to the development of frailty, like any other comorbid disease18, rather than a global underlying condition of vulnerability. This might lead to the conclusion that older adults recently diagnosed with cancer and who have not received yet the cancer treatment do not differ from non-cancer patients in terms of frailty level and even global health status (as illustrated by the frailty index (FI), which is based on deficit accumulation).

Our findings are somewhat surprising, since multiple types of cancer in the elderly are known to be associated with weight loss (7), as corroborated by our findings, but also reduced physical activity (19), muscle weakness (20), and fatigue (21), all of them composing the frailty phenotype. However, this was not the case in our study, suggesting that older subjects with a recent diagnosis of cancer have similar health status, as illustrated by both the frailty phenotype and the FI, than same-age peers receiving the same healthcare services to fight against frailty. Therefore, it is plausible to think that the bad prognosis associated to cancer reflects disease- and/or cancer treatment-related burden rather than an underlying global status of frailty.

Furthermore, the absence of exhaustion, weakness, slow gait difference and low physical activity among the two groups doesn’t exclude its evitability in managing cancer patients for frailty and pre-cancer treatment but illuminate the idea that those clinical anomalies might not be phenotypically expressed and aggravated before malnutrition occurs or cancer evolution. Nutrition disorders are highly prevalent among patients with advanced cancer and can lead to reduced quality of life and poorer treatment outcome (7) leading to sarcopenia and cancer cachexia. Thus, intervening to reverse the cycle can be primordial so that dealing with those cancer patients could be similar in frailty management compared to those without cancer. Another hypothesis is that the probable evolution of the phenotype and its divergence between the two groups is much more relying on the fundamentals of aging, gender risk and the overlapping influence of the comorbidity (i.e.: cancer) rather than frailty risk factors (22).

Strength of this cross-sectional study included measurement of a validated construct of frailty and its comparison between two groups with large sample size. Furthermore, our study included a real-life population that came to the same frailty clinic for an evaluation whether they have or not cancer. However, this cross-sectional study impeded us of examining if frail older adults are at an increased risk of functional decline after cancer treatment and the burden of treatment on the evolution of frailty over time. This study did not show any association of cancer with frailty levels in a sample of older patients receiving ambulatory care against frailty. Cancer patients should receive a similar frailty management than people without cancer, at least, in the pre-cancer treatment phase.

## Notes

*Ethical standards:* Patient’s data and results were treated as part of their medical evaluation at the Geriatric Frailty Clinic (GFC), in the Toulouse University Hospital, France. Patients were referred by their primary care physician for frailty assessment or by their oncologist for care plan management including pre-cancer treatment. Furthermore patients were asked for their approval before entering data for research purposes.
